# *SNZ3* Encodes a PLP Synthase Involved in Thiamine Synthesis in *Saccharomyces cerevisiae*

**DOI:** 10.1534/g3.118.200831

**Published:** 2018-11-29

**Authors:** Michael D. Paxhia, Diana M. Downs

**Affiliations:** Department of Microbiology, University of Georgia, Athens, GA 30602

**Keywords:** *SNZ*, *SNO*, PLP synthase, PLP biosynthesis, thiamine biosynthesis

## Abstract

Pyridoxal 5′-phosphate (the active form of vitamin B_6_) is a cofactor that is important for a broad number of biochemical reactions and is essential for all forms of life. Organisms that can synthesize pyridoxal 5′-phosphate use either the deoxyxylulose phosphate-dependent or -independent pathway, the latter is encoded by a two-component pyridoxal 5′-phosphate synthase. *Saccharomyces cerevisiae* contains three paralogs of the two-component *SNZ/SNO* pyridoxal 5′-phosphate synthase. Past work identified the biochemical activity of Snz1p, Sno1p and provided *in vivo* data that *SNZ1* was involved in pyridoxal 5′-phosphate biosynthesis. Snz2p and Snz3p were considered redundant isozymes and no growth condition requiring their activity was reported. Genetic data herein showed that either *SNZ2* or *SNZ3* are required for efficient thiamine biosynthesis in *Saccharomyces cerevisiae*. Further, *SNZ2* or *SNZ3* alone could satisfy the cellular requirement for pyridoxal 5′-phosphate (and thiamine), while *SNZ1* was sufficient for pyridoxal 5′-phosphate synthesis only if thiamine was provided. qRT-PCR analysis determined that *SNZ2,3* are repressed ten-fold by the presence thiamine. In total, the data were consistent with a requirement for PLP in thiamine synthesis, perhaps in the Thi5p enzyme, that could only be satisfied by *SNZ2* or *SNZ3*. Additional data showed that Snz3p is a pyridoxal 5′-phosphate synthase *in vitro* and is sufficient to satisfy the pyridoxal 5′-phosphate requirement in *Salmonella enterica* when the medium has excess ammonia.

Pyridoxal-5′-phosphate (PLP, the active form of vitamin B_6_) is an essential cofactor that is used for diverse reactions including α/β eliminations, retro-aldol cleavages, transaminations and racemizations (Toney 2011). Two pathways for PLP biosynthesis have been described. The 1-deoxy-D-xylulose-5-phosphate (DXP)-independent pathway is found in most bacteria, archaea and eukaryotes, while a DXP-dependent pathway is found in some bacteria including the proteobacteria, firmicutes, chlorobi, cyanobacteria and aquificae (Tanaka *et al.* 2005; Fitzpatrick *et al.* 2007; Mukherjee *et al.* 2011; Rosenberg *et al.* 2017). The DXP-dependent pathway synthesizes PLP from erythrose-4-phosphate, glyceraldehyde-3-phosphate and pyruvate in a series of seven enzymatic steps. Formation of the pyridine heterocyclic ring in this pathway is catalyzed by the pyridoxine-5′-phosphate synthase (E.C. 2.6.99.2), which is encoded by *pdxJ* in *Escherichia coli* (Figure 1). In contrast, the DXP-independent pathway uses two enzymes to create the heterocyclic pyridine ring of PLP from glutamine, glyceraldehyde-3-phosphate and ribose-5-phosphate (Mukherjee *et al.* 2011) (Figure 1). Salvage of B_6_ vitamers requires enzymes that are conserved across organisms. Notably, pyridoxine phosphate oxidase (PNPO, PdxH; E.C.:1.4.3.5) is required for *de novo* synthesis in the DXP-dependent pathway and is required for salvage in organisms using either pathway.

**Figure 1:**
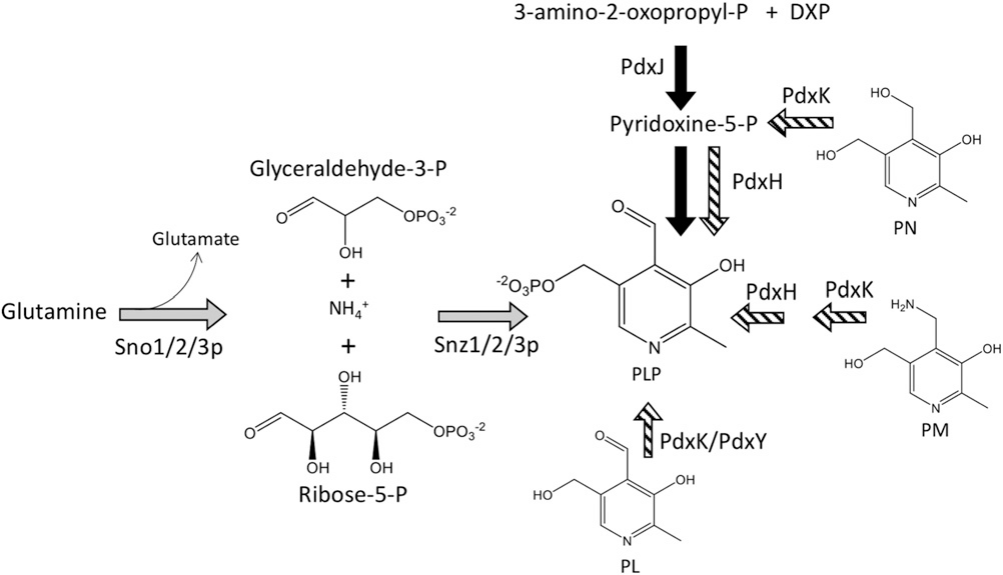
Formation and modification of the pyridine heterocycle in PLP biosynthesis. Two biosynthetic strategies for the formation of the pyridine heterocycle of PLP are depicted. The final two steps of the DXP-dependent pathway for PLP biosynthesis are shown in black arrows, and the DXP-independent pathway is shown with gray. Enzymatic steps involved in salvage of B_6_ vitamers are depicted with hashed arrows. Proteins catalyzing each step are noted by the relevant arrows, using *E. coli* (black/hatched) or *S. cerevisiae* (gray) nomenclature. Abbreviations: DXP – deoxyxyulose-5-phosphate; PL – pyridoxal; PM – pyridoxamine; PN – pyridoxine; PLP – pyridoxal-5′-phosphate.

The two enzymes unique to the DXP-independent pathway to PLP form a complex comprised of a glutaminase and a PLP synthase subunit (EC 4.3.3.6) (Burns *et al.* 2005; Raschle *et al.* 2005; Strohmeier *et al.* 2006). In general, the glutaminase subunit liberates ammonia from glutamine and delivers it to the PLP synthase subunit, where it combines with glyceraldehyde-3-phosphate and ribose-5-phosphate to form PLP (Burns *et al.* 2005; Raschle *et al.* 2005; Strohmeier *et al.* 2006). Similar ammonia tunneling is a feature of several multi-subunit synthase enzymes that use glutamine as a source of ammonia including, carbamoyl-phosphate synthetase (E.C. 6.3.5.5), anthranilate synthase (E.C. 4.1.3.27), aminodeoxychorismate synthase (E.C. 2.6.1.85) and imidazole glycerol phosphate synthase (E.C. 4.3.2.-) (Makoff and Radford 1978; Klem and Davisson 1993; Romero *et al.* 1995; Viswanathan *et al.* 1995). In cases where it has been tested, the glutaminase subunit is dispensable both *in vitro* and *in vivo* when there are high levels of ammonia (Huang *et al.* 2001; Belitsky 2004).

Several PLP synthase enzymes from bacteria, archaea, yeast and plants, have been characterized biochemically with and without the associated glutaminase (Dong *et al.* 2004; Burns *et al.* 2005; Raschle *et al.* 2005; Gengenbacher *et al.* 2006; Strohmeier *et al.* 2006; Zein *et al.* 2006; Raschle *et al.* 2007; Hanes *et al.* 2008; Neuwirth *et al.* 2009; Raschle *et al.* 2009; Moccand *et al.* 2011; Robinson *et al.* 2016; Rodrigues *et al.* 2017). A variety of names have been used for the genes encoding PLP synthase and glutaminase enzymes, *e.g.*, *SNZ/pdxS/pdx1*, *SNO/pdxT/pdx2*, respectively. The varied nomenclature has complicated the analyses and comparison of these enzymes across organisms. For simplicity the names for the glutaminase and synthase proteins coined in yeast, Sno and Snz, respectively are used herein. Organisms can encode multiple paralogs of *SNZ* and *SNO*, and in most cases the value of the redundancy remains unclear (Rodríguez-Navarro *et al.* 2002; Tambasco-Studart *et al.* 2005; Titiz *et al.* 2006; Boycheva *et al.* 2015; Moccand *et al.* 2014; Dell’Aglio *et al.* 2017). An exception is *Arabidopsis thaliana*, where each of the three isozymes of Snz have been characterized *in vivo* and *in vitro* (Titiz *et al.* 2006; Moccand *et al.* 2014). Two of the isozymes (Pdx1.1, Pdx1.3) had PLP synthase activity *in vitro*, and either was sufficient for PLP synthesis *in vivo* (Moccand *et al.* 2014). The third isozyme (Pdx1.2) had weak homology, lacked key residues involved in catalysis, and failed to generate a detectable phenotype when absent (Moccand *et al.* 2014).

*Saccharomyces cerevisiae* has three glutaminase paralogs (*SNO1-SNO3*) and three PLP synthase paralogs (*SNZ1-SNZ3*) (Rodríguez-Navarro *et al.* 2002). *SNZ1* was required for growth in the absence of exogenous pyridoxine, but the need for *SNO1 in vivo* was less clear (Rodríguez-Navarro *et al.* 2002; Stolz and Vielreicher 2003). Snz1p is a PLP synthase that uses glyceraldehyde-3-phosphate, ribose-5-phosphate and free ammonia as substrates *in vitro* (Neuwirth *et al.* 2009), and Sno1p has glutaminase activity *in vitro* (Dong *et al.* 2004). The inability of an *snz1* mutant to grow on dropout medium lacking PN was attributed to the presence of thiamine. The *SNZ2/3* genes were regulated by thiamine, in a manner completely dependent on *THI2* and partially dependent on *THI3* (Rodríguez-Navarro *et al.* 2002). The pattern of regulation suggested *SNZ2/3* could have a role in thiamine synthesis and/or metabolism, although to our knowledge this idea was not directly tested experimentally.

A long-time interest in metabolic network structure and robustness, and redundancy in vitamin biosynthesis, with a focus on thiamine (reviewed in (Downs 2006; Koenigsknecht and Downs 2010)), prompted us to explore the functional roles of the Sno/Snz proteins in *S. cerevisiae*. Herein we assimilate the genetic and biochemical characterization of the *SNZ* paralogs to lay the groundwork for future work on the integration of the biosynthesis of two essential cofactors, thiamine and PLP, in *Salmonella enterica* and *S. cerevisiae*. The data presented confirmed that *SNZ3* encodes a PLP synthase, quantified the transcriptional regulation of *SNZ2* and *SNZ3* by thiamine, and showed that strains lacking *SNZ2* and *SNZ3* required thiamine supplementation for growth. Together these data provide the first report of a functional role for these genes *in vivo*.

## Materials and Methods

### Strains, Media and Chemicals

#### Yeast:

*S. cerevisiae* strains used in this work were derived from YJF153 (*MATa HO*::*dsdAMX4*, a haploid derivative of YPS163) (Li and Fay 2017), and the relevant genotypes are listed in Table 1. *S. cerevisiae* strains were routinely grown on rich medium containing 10 g/L yeast extract, 20 g/L peptone, 20 g/L dextrose and 20 g/L agar (YPD). Two variations on defined medium were used to monitor vitamin requirements. Synthetic defined media (SD, SG) contained 1.7 g/L yeast nitrogen base without amino acids or nitrogen (YNB, Sunrise Science catalog no. 1500-100) or the respective drop-out as indicated (YNB-Pyridoxine, YNB-Thiamine, YNB-Pyridoxine-Thiamine) (Sunrise Science), 5 g/L ammonium sulfate, 20 g/L agar and either 20 g/L dextrose or 30 g/L glycerol as carbon source.

**Table 1:** – Strains, Plasmids and Primers

Strain Number	Genotype
*Saccharomyces cerevisiae*	
YJF153	WT
DMy49	*snz1*::kanMX-loxP
DMy51	*snz1*::kanMX-loxP *snz3*::hphMX-loxP
DMy52	*snz1*::kanMX-loxP *snz2*::natMX-loxP *snz3*::hphMX-loxP
DMy53	*snz2*::kanMX-loxP
DMy54	*snz2*::kanMX-loxP *snz3*::natMX-loxP
DMy55	*sno1*::kanMX-loxP
DMy56	*snz1*::kanMX-loxP *snz2*::hphMX-loxP
DMy57	*snz3*::kanMX-loxP
*Salmonella enterica*	
DM7080	Δ*araCBAD*
DM15839	Δ*araCBAD pdxJ662*::Kn / pSU18
DM15840	Δ*araCBAD pdxJ662*::Kn / pDM1595
DM15843	Δ*araCBAD pdxJ662*::Kn / pDM1595 pBAD24
DM15844	Δ*araCBAD pdxJ662*::Kn / pDM1595 pDM1596
**Plasmid Name**	**Description or Reference**
pSU18	(Bartolomé *et al.* 1991)
pBAD24	(Guzman *et al.* 1995)
pDM1595	pSU18-*SNZ3*
pDM1596	pBAD24-*SNO3*
**Primer Name**	**Sequence**
CM	CCTCGACATCATCTGCCC
AGP3R	CGTTCCAGAATAGAAGGTCGA
RPD3R	TGTCAACTATGCGGGTGGTTT
pdxJ F	AACGCACAGTAAAAACGAAGAAAGATTAACGAGGATTGTCGTGTAGGCTGGAGCTGCTTC
pdxJ R	GGGCAATCTCTACAATATCCGTTCCCAGGCCGAGAATCGCCATATGAATATCCTCCTTAG
5′ SNZ2 *Sac*I	TAGGGAGCTCAGGAGGACAGCT**ATG**TCAGAATTCAAGGTTAAAACTG
3′ SNZ2 *Xba*I	TAGGTCTAGACTACCATCCGATTTCAGAAAGTC
SNZ1 F	AGTAAATATACACAGTACTAATATTCAGTTAATTATCACGCAGCTGAAGCTTCGTACGC
SNZ1 R	GGAAAAGTGTTATAATGCTCAAAATACCTGTTCAAAGAAAGCATAGGCCACTAGTGGATCTG
SNZ2 F	ACTATAATAGAAAAATAAGTATATCGTAAAAAAGACAAAACAGCTGAAGCTTCGTACGC
SNZ2 R	TCGAAGGAAACAAATTAGCGTTGTGTGAGCATCGCTAGTTGCATAGGCCACTAGTGGATCTG
SNO1 F	TTCATTTCGTTAAATAGAAAGAAAAACCATATCTTAAAGTCAGCTGAAGCTTCGTACGC
SNO1 R	AGGTTTTGGTAATATAAAAATGTGGAAAACCGGCGGTATTGCATAGGCCACTAGTGGATCTG
5′ SNO2 *Nco*I 2	TAGGACC**ATG**GCCGTCGTTATCGGAGT
3′ SNO2 *Pst*I 2	TAGGCTGCAGAGGCGAGTTCAGAATGAACA
5′ SNZ2 *Nhe*I	TAGGGCTAGCATGTCAGAATTCAAGGTTAAAACTG
3′ SNZ2 *Nco*I	TAGGCCATGGCTACCATCCGATTTCAGAAAGTC
ALG9 qRT-PCR F	TCACGGATAGTGGCTTTGGT
ALG9 qRT-PCR R	CATTCACTACCGGTGCCTTC
UBC6 qRT-PCR F	ATCCTGGCTGGTCTGTCTCA
UBC6 qRT-PCR R	ATTGATCCTGTCGTGGCTTC
SNZ2/3 qRT-PCR F	GCAATGATCCGTACCAAAGG
SNZ2/3 qRT-PCR R	CCGCCTTAATCTTGGTGATG

Underlining identifies an added ribosome binding site (RBS), bold letters represent start codon.

A minimal medium (Minimal Vitamin Dextrose; MVD) contained 1.7 g/L YNB-Vitamins (Sunrise Science), biotin (0.002 mg/L), and D-pantothenic acid hemicalcium salt (0.4 mg/L), 5 g/L ammonium sulfate, 20 g/L agar and 20 g/L dextrose as carbon source. Thiamine (0.4 mg/L) and/or pyridoxine (0.4 mg/L) were added as indicated. Antibiotics used for deletion marker selection were added to the following final concentrations in YPD: 400 mg/L geneticin (G-418 sulfate), 200 mg/L Hygromycin B, and 100 mg/L nourseothricin sulfate (clonNAT) (Gold Biotechnology). A lower concentration of 200 µg/mL geneticin was used for maintenance of strains with G-418 resistance. 2-methyl-4-amino-5-hydroxymethylpyrimidine (HMP) was purchased from LabSeeker, Inc.

#### Bacteria:

Media for bacterial growth were Nutrient Broth (NB) containing 8 g/L Difco Nutrient broth and 5 g/L NaCl, lysogeny broth (LB), or superbroth (SB; 32 g/L tryptone (Fisher Scientific), 20 g/L yeast extract (Fisher Scientific), 5 g/L NaCl with 0.05 N NaOH). Solid media contained 1.5% agar. Antibiotics were added at the following concentrations in rich media, unless otherwise indicated: kanamycin (Kn), 50 mg/L; chloramphenicol (Cm), 20 mg/L; ampicillin (Ap), 100 mg/L. Minimal media was no-carbon E medium (NCE) (Vogel and Bonner 1956) with 1 mM MgSO_4_, 0.1x trace minerals (Balch *et al.* 1979), with either glucose (11 mM) or glycerol (22 mM) (Fisher Scientific) as a sole carbon source. Minimal medium with low nitrogen was no-carbon and nitrogen (NCN) (Davis *et al.* 1980) with 1 mM glutamine and glucose or glycerol as sole carbon source. All strains of *S. enterica* are derived from strain LT2 and their relevant genotypes are described in Table 1. Chemicals were purchased from Sigma-Aldrich, St. Louis, MO unless otherwise indicated.

### Genetic Techniques

In-frame deletions of genes in *S. enterica* were created with Lambda-Red recombineering as described (Datsenko and Wanner 2000). Insertions were reconstructed by transduction into DM7080 (*araCBAD*) with the high-frequency generalized transducing mutant of bacteriophage P22 (HT105/1, int-201) (Schmieger 1972). Primers used to generate these deletions are listed in Table 1.

Gene disruptions in *S. cerevisiae* were made using a described gene replacement method (Hegemann and Heick 2011). Antibiotic cassettes were amplified from the appropriate plasmid using primers listed in Table 1. Five µg of purified DNA was transformed into *S. cerevisiae* by incubating cells suspended in a mixture of 33% polyethylene glycol 3350 (PEG 3350), 100 mM lithium acetate, and 0.28 mg/mL salmon sperm DNA at 42° for 90 min. The transformed cells were recovered in YPD for 3 hr with shaking at 30° and plated to YPD with the appropriate antibiotic. Colonies that grew on the plates after three days were streaked onto selective media and insertions were confirmed by colony PCR. Insertions in *SNZ2* and *SNZ3* were distinguished by PCR using gDNA as a template and AGP3R, RPD3R, and CM primers listed in Table 1.

### Molecular Techniques

Plasmids were constructed using standard molecular techniques. Plasmid DNA was isolated using the PureYield Plasmid MiniPrep System (Promega, Madison, WI). Q5 DNA polymerase (New England Biolabs, Ipswich, MA) was used to amplify DNA with primers synthesized by Integrated DNA Technologies, Coralville, IA or Eton Bioscience, Inc., Research Triangle Park, NC. PCR products were purified using the PCR purification kit (Qiagen, Venlo, Limburg, The Netherlands). Restriction endonucleases were purchased from New England Biolabs, Ipswich, MA, and ligase was purchased from ThermoScientific, Waltham, MA.

### Growth Analysis

#### Bacteria:

Growth of *S. enterica* strains were monitored at OD_650_ in 96 well plates with a BioTek ELx808 plate reader. Strains were grown overnight in NB with Cm or Ap as indicated and inoculated at 1% into 200 µL of media indicated. Plates were incubated at 37° with medium shaking and data were plotted using Prism 7 (GraphPad).

#### Yeast:

Growth of *S. cerevisiae* strains was followed by dilution plating or liquid growth. Liquid growth was monitored at OD_650_ in 96 well plates with a BioTek ELx808 plate reader. Strains were grown for 24 hr in SD before pelleting and resuspending twice in saline. Washed cells were inoculated in 100 µL of the appropriate medium at 1%. Plates were incubated at 30° with fast shaking and data were plotted using Prism 7 (GraphPad).

To monitor growth via dilution plating, *S. cerevisiae* strains were grown for 24 hr in YPD before pelleting, resuspending in saline, and 5 µL of serial dilutions in saline from 10^−2^ to 10^−7^ were plated onto the respective media. Plates were incubated at 30° for two or three days, as indicated.

### Reverse transcription-quantitative-PCR

RNA from three biological replicates of YJF153 was prepared and extracted as follows. Independent cultures of YJF153 were grown for 24 hr in SD with shaking at 30° before pelleting and resuspending twice in saline. Washed cells were used as a 1% inoculum into 5 mL MVD with and without thiamine and/or PN as indicated and grown at 30° with shaking for 12 hr. Cells were pelleted at 10,000×g for 15 sec and frozen in liquid nitrogen. RNA was extracted using the RNA*snap* method modified for yeast (Stead *et al.* 2012). Briefly, 100 µL of glass beads (425-600 µm) and 110 µL of RNA extraction solution (95% molecular biology grade formamide, 0.025% SDS, 18 mM EDTA and 1% β-mercaptoethanol) were added to the cell pellets and vortexed with a bead-beating adaptor for five minutes. Tubes were then incubated at 95° for 7 min and centrifuged at 16,000×g for 5 min. The supernatant was transferred into another tube and RNA was concentrated by sodium acetate/ethanol precipitation, treated with RNase-free Turbo DNase (Ambion), precipitated by sodium acetate/ethanol precipitation and stored at -80°.

Quality and concentration of total RNA was evaluated at the Georgia Genomics and Bioinformatics Core (GGBC) using the RNA nano 6000 kit for the Agilent Bioanalyzer 2100. cDNA was generated from 800 ng of total RNA using the iScript cDNA synthesis kit (Bio-Rad Laboratories) by following the manufacturer’s protocol. Real-time PCR reactions (20 µL) were prepared with 10 µL Fast SYBR Green Master Mix (Applied Biosystems), 8 ng cDNA and 500 nM gene-specific primers (Table 1). Real-time PCR was performed using the Applied Biosystems 7500 Fast real-time PCR system. Expression of *SNZ2* and *SNZ3* were treated as one transcriptional response, due to their 99% identity at the nucleotide level. Relative expression of *SNZ2/3* was calculated using the comparative cycle threshold method (ΔΔC_T_) with *UBC6* as an internal control and fold change (treated/untreated) was calculated with the equation 2^−ΔΔC_T_^ (Teste *et al.* 2009; Livak and Schmittgen 2001). The standard error of the mean (SEM) was calculated for the test condition ΔC_T_, using Guassian error propagation, and this was used to calculate the 95% confidence intervals for each ΔΔC_T_ calculation. To ensure the effectiveness of using *UBC6* as an internal standard, *ALG9* was used as an alternative internal control (Teste *et al.* 2009). Under the conditions tested, no significant differences in expression were observed for either internal control.

### Protein purification

*SNZ3* was cloned into pTEV5 (Rocco *et al.* 2008) at the *Nhe*I/*Nco*I sites, the plasmid was purified and transformed into *E. coli* BL21-AI. The resulting strain was grown overnight at 37° in 100 mL NB Ap and inoculated into six liters of SB Ap (1%), and grown at 37° with shaking (200 rpm) to an OD_650_ of 0.6. The temperature was lowered to 30°, arabinose added to a final concentration of 0.2% and cells were incubated for 19 hr prior to harvesting by centrifugation. The cell pellet was resuspended in Buffer A (50 mM HEPES, 300 mM NaCl, 20 mM Imidizole, pH 7.5 at 4°) with DNAse (0.025 mg/mL), lysozyme (1 mg/mL) and phenylmethylsulfonyl fluoride (0.1 mg/mL) and kept on ice for one hour. The cell suspension was lysed at 20 kpsi using a Constant Systems Limited One Shot (United Kingdom), and cell lysate was cleared at 48,000 xg for 50 min at 4°. The cell-free extract was passed through a 0.45 µM PVDF filter (Millipore) and injected onto a pre-equilibrated 5 mL HisTrap HP Ni-sepharose column. The column was washed with 5 column volumes of Buffer A, followed by 5 column volumes of 4% Buffer B (50 mM HEPES, 300 mM NaCl, 500 mM Imidazole, pH 7.5 at 4°) and finally a gradient of Buffer B from 4 to 100% over 10 column volumes. Fractions containing Snz3p were combined, rTEV protease was added at a 50:1 protein to rTEV ratio and the mixture sat for 3.5 hr at room temperature before it was dialyzed into Buffer A overnight at 4° with 3 buffer changes. The tagless protein was separated from His_6_-Snz3p and His_6_-rTEV by gravity column chromatography with HisPur Ni-NTA resin. Snz3p was concentrated by centrifugation using a 10 kD filter (Millipore), exchanged into a 50 mM HEPES buffer, pH 7.5, with 10% glycerol using a PD10 column (GE Healthcare), flash-frozen in liquid nitrogen and stored at -80° until use. Protein concentration was determined by bicinchoninic acid (BCA) assay (Pierce) with bovine serum albumin as a standard. The Snz3p preparation was > 98% pure based on densitometery.

### PLP synthase assay

Snz3p was thawed and dialyzed into assay buffer (50 mM Tris-HCl, pH 8.0). Reactions were performed in a buffer of 50 mM Tris-HCl, pH 8.0 at 37° with ammonium sulfate (10 mM), D/L glyceraldehyde-3-phosphate, ribose-5-phosphate and Snz3p (Raschle *et al.* 2005; Neuwirth *et al.* 2009). Assays were performed in triplicate with 85 µM Snz3p and the formation of PLP was followed spectrophotometrically at 414 nm using Spectramax 398-Plus plate reader. The extinction coefficient for PLP in assay buffer was determined to be 7.57 × 10^3^ M^-1^ cm^-1^ at 414 nm with a five-point standard curve measured in duplicate from 0 – 0.1 M PLP (R^2^ = 0.9996). Kinetic parameters for Snz3p with D/L glyceraldehyde-3-phosphate as a substrate were determined with 1 mM ribose-5-phosphate and reactions were initiated with concentrations of D/L glyceraldehyde-3-phosphate from 62.5 µM to 2 mM. Activity of Snz3p with ribose-5-phosphate as a substrate was determined with 2 mM D/L glyceraldehyde-3-phosphate and reactions were initiated with concentrations of ribose-5-phosphate from 25 µM to 0.8 mM. Data were plotted and analyzed using Prism 7 (Graph Pad).

### Data Availability

Strains and plasmids are available upon request. The authors affirm that all data necessary for confirming the conclusions of the article are present within the article, figures, and tables.

## Results and Discussion

### SNZ1, 2, 3 paralogs have distinguishable roles in vivo

Mutants of *S. cerevisiae* YJF153 lacking one or more of the *SNZ* paralogs were constructed to evaluate the role of these proteins *in vivo* (Table 1). The YJF153 strain of *S. cerevisiae* was chosen for use because it i) has a wild type allele of the transcriptional regulator *THI3*, and ii) has no auxotrophy. The former point is relevant in that the status of *THI3* impacts expression of genes in the thiamine regulon, which includes *SNZ2* and *SNZ3*, due to its activity as a co-activator with Thi2p (Rodríguez-Navarro *et al.* 2002; Nosaka *et al.* 2005; Mojzita and Hohmann 2006; Brion *et al.* 2014). The standard laboratory strain of *S. cerevisiae* (S288C) has a mutant allele of *THI3* which results in abhorrent expression of the thiamine regulon (Brion *et al.* 2014). The lack of any auxotrophy indicated the strain had the functional metabolic network needed to dissect metabolic interactions and detect often subtle connections between pathways.

**Figure 2:**
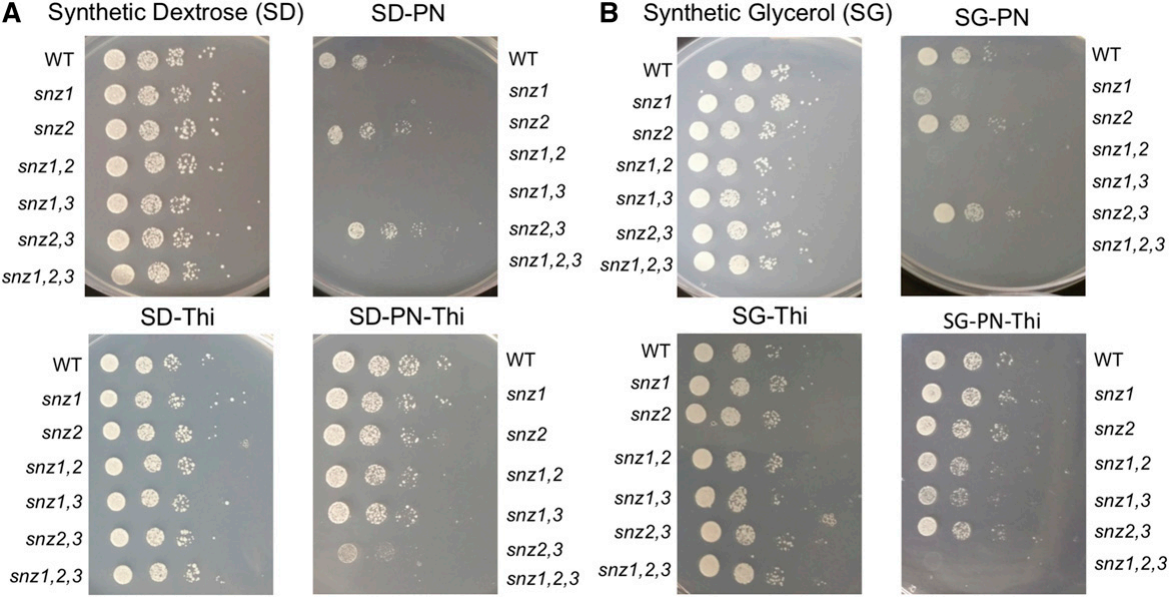
*SNZ1* is required for PLP synthesis in the presence of thiamine. Strains with deletions of *SNZ1*, *SNZ2*, *SNZ1,2*, *SNZ1,3*, *SNZ2,3* and *SNZ1,2,3* were grown overnight in YPD and 5 µL each of dilutions from 10^−2^ to 10^−7^ were spotted on synthetic dextrose (A) or glycerol (B) media or a dropout derivative of this medium lacking pyridoxine and/or thiamine as indicated. Plates were incubated for two (SD) or three (SG) days at 30 °C. Growth of the strain deleted for *SNZ3* was indistinguishable from the *snz2* mutant.

Eight strains (single, double and triple mutants) were constructed to query the role of the *SNZ* paralogs. Standard yeast drop-out media with glucose or glycerol were used and the data for seven of the mutants are shown in Figure 2. Growth of an *snz3* strain was indistinguishable from the *snz2* strain under these conditions (data not shown). Consistent with previous observations, *SNZ1* was required for growth in dextrose medium lacking only pyridoxine (SD-PN). However, when thiamine was also excluded (SD-PN-Thiamine), a single functional copy of any of the *SNZ* paralogs allowed growth. These data were consistent with a model where each PLP synthase had the capacity to generate sufficient PLP for growth, but the regulation of *SNZ2,3* by thiamine prevented them from contributing to PLP synthesis in its presence. The expression of *SNZ2* or *SNZ3 in trans* had been shown to complement an *snz1* mutant, but this is the first demonstration that at chromosomal levels, either *SNZ2* or *SNZ3* was sufficient for PLP synthesis that allowed optimal growth.

A similar analysis was done on media with glycerol rather than dextrose to compare fermentative *vs.* respiratory lifestyles (Figure 2B). The data were generally similar, with two differences noted. First, deletion of *SNZ1* severely decreased, but did not eliminate growth on SG-PN medium. These data suggested that either the PLP requirement was lower during glycerol respiration, or the repression of *SNZ2,3* in the presence of thiamine was weaker on glycerol. Second, we noted that the *snz2,3* double mutant grew poorly on SD-PN-Thiamine, while it showed robust growth on SG-PN-thiamine, suggesting *SNZ1* was not always sufficient for PLP synthesis.

#### Minimal vitamin medium clarifies the role of SNZ2 and SNZ3:

**Figure 3:**
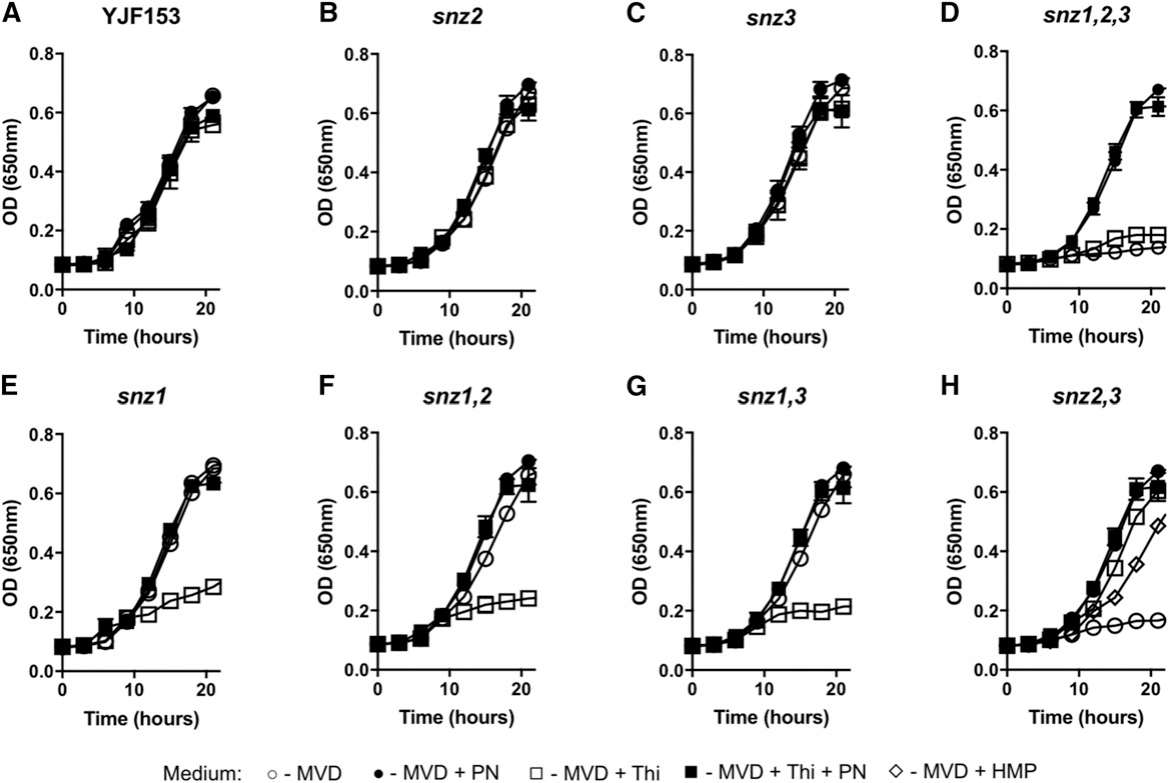
*SNZ2* or *SNZ3* is required for growth on minimal dextrose media. Growth of wildtype *S. cerevisiae* (A) and strains with deletions of *SNZ2* (B), *SNZ3* (C), *SNZ1,2,3* (D), *SNZ1* (E), *SNZ1,2* (F), *SNZ1,3* (G), or *SNZ2,3* (H) was monitored on minimal vitamin media with dextrose (MVD; circles). The strains were also grown in MVD with added PN (filled symbols) and/or thiamine (squares), and the legend is shown. In the case of the *snz2,3* mutant, growth in MVD with added HMP (200 nM) is also shown (diamonds). Error bars indicate the standard deviation of three independent biological replicates.

**Figure 4:**
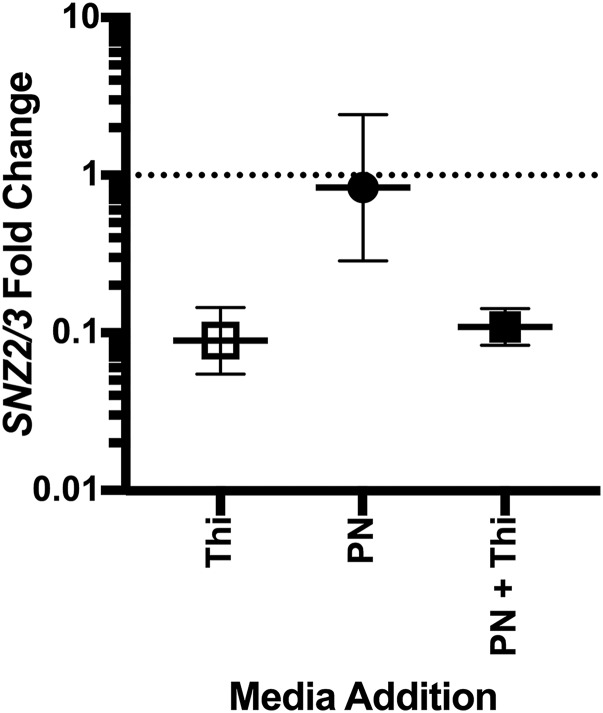
Expression of *SNZ2/3* is repressed by thiamine. Expression of *SNZ2* and *SNZ3* was determined by qRT-PCR when YJF153 was grown for twelve hours in MVD medium with various additions. Fold change represents the ratio of expression on MVD supplemented with thiamine (0.4 mg/L), pyridoxine (0.4 mg/L), or both (as indicated) compared to the expression on MVD with no supplements. Error bars indicate the 95% confidence interval of three independent biological replicates.

It was formally possible that additional nutrients in the drop out medium were complicating the interpretation of the nutritional phenotypes due to unanticipated metabolic interactions or regulation. The results above were readdressed using medium with supplementation, rather than drop-out. Control experiments showed that *S. cerevisiae* strain YJF153 grew well on synthetic dextrose medium with YNB-vitamins if both biotin and pantothenate provided. This medium was designated as Minimal Vitamin Dextrose medium (MVD) and used in the subsequent experiments. The growth of the eight strains described above was quantified in liquid MVD, with pyridoxine and/or thiamine added exogenously (Figure 3). Several points were taken from the resulting data. The parental wild type strain (YJF153), DMy53 (*snz2*), and DMy57 (*snz3*) strains had full growth on each medium (Figure 3A,B,C), while the triple mutant (DMy52) failed to grow in the absence of PN (Figure 3D). The other three strains that lacked *SNZ1* grew in MVD, supporting the conclusion that either isozyme was sufficient for PLP synthesis in the absence of repression by thiamine. However, the strains that depended on *SNZ2* and/or *SNZ3* for PLP synthesis, failed to grow if thiamine was present in the medium (Figure 3 E,F,G). To verify the explanation that the lack of growth was due to transcriptional repression caused by thiamine, transcript levels of *SNZ2* and *SNZ3* were determined by qRT-PCR. The relative expression level of *SNZ2/3* in YJF153 grown in MVD compared to MVD containing thiamine, PN, or both was measured. The data in Figure 4 showed that the addition of thiamine repressed transcription of *SNZ2/3* approximately 10-fold. The presence of PN did not affect this repression and had no detectable effect by itself. These data validated the model that the conditional auxotrophy of strains lacking *SNZ1* in the presence of thiamine is due to the transcriptional repression of *SNZ2* and *SNZ3*.

The data in Figure 3 shed new light on the role and limitation of *SNZ1* function that was only hinted at by the data in Figure 2. The *snz2,3* double mutant failed to grow on MVD medium (Figure 3H). These data showed the PLP synthase encoded by *SNZ1* was not able to provide sufficient PLP synthesis for growth. Growth was restored by the addition of either thiamine or pyridoxine. The growth behavior of the *snz2,3* double mutant was consistent with a scenario in which thiamine synthesis required PLP and Snz1p alone could not synthesize sufficient PLP to satisfy this requirement. In fact, in *S. cerevisiae*, synthesis of the 2-methyl-4-amino-5-hydroxymethylpyrimidine (HMP) moiety of thiamine involves use of PLP as a substrate by a poorly characterized HMP-P synthase enzyme, Thi5p (Wightman and Meacock 2003). Consistently, exogenous addition of HMP (but not the thiazole moiety) restored growth to *snz2,3* double mutant. Together, these data suggest the synthesis of thiamine, potentially via Thi5p, has unique PLP requirements which could help explain the presence of multiple *SNZ/SNO* paralogs in *S. cerevisiae*.

### SNO1 is required for PLP synthesis when ammonia is limiting in vivo

**Figure 5:**
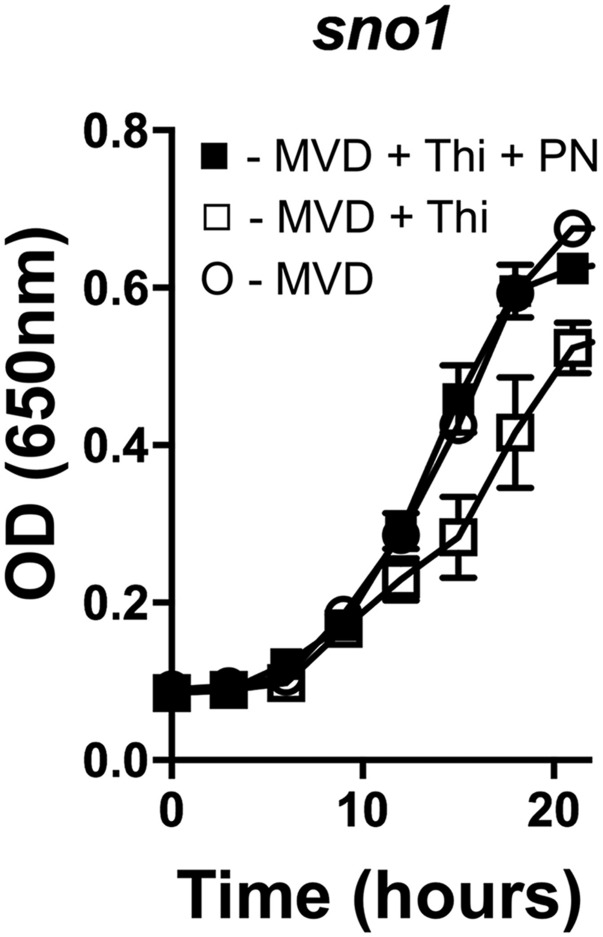
*SNO1* contributes to PLP biosynthesis in *S. cerevisiae*. *A sno1* mutant was grown on minimal vitamin dextrose medium (open circles), with addition of thiamine (open squares), and with addition of thiamine and pyridoxine (filled squares). Error bars indicate the standard deviation of three independent biological replicates.

In *S. cerevisiae* each *SNZ* paralog has a corresponding *SNO* glutaminase subunit (Rodríguez-Navarro *et al.* 2002). The need for the glutaminase activity in PLP synthesis has not been clearly demonstrated *in vivo* (Rodríguez-Navarro *et al.* 2002; Stolz and Vielreicher 2003). The role of *SNO1* on MVD was tested (Figure 5). The strain lacking *SNO1* had a small but reproducible growth defect when thiamine was added, that was corrected with the addition of PN. This result suggested that *SNO1* was required for optimal PLP synthesis. This interpretation was complicated by the presence of other *SNO* paralogs, despite the assumption their expression was completely repressed by the thiamine (Rodríguez-Navarro *et al.* 2002).

**Figure 6:**
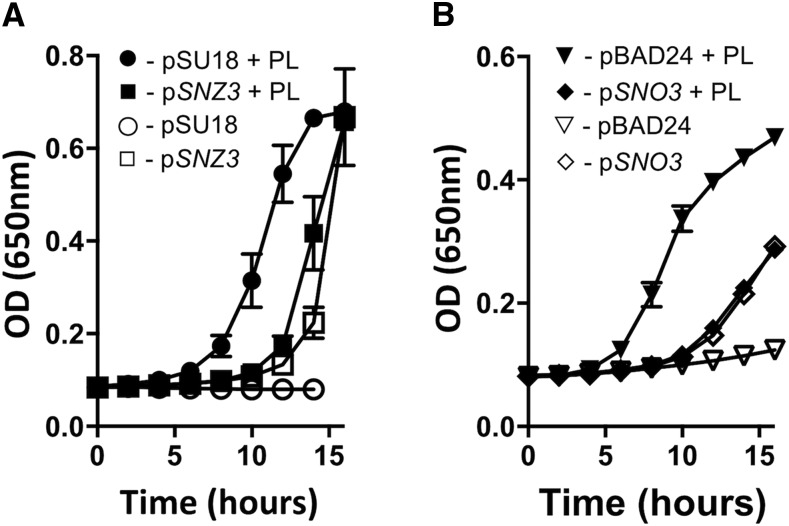
*SNZ3* and *SNO3* can synthesize PLP in *Salmonella enterica*. Growth of *pdxJ* mutant of *S. enterica* with various plasmids was monitored for growth. In panel (A) the *pdxJ* mutant carried an empty vector (pSU18) (circles) or a plasmid expressing *SNZ3* (squares). Each of these two strains were grown on NCE (*i.e.*, high ammonia) minimal medium with glycerol. Filled and open symbols represent growth in the absence, while solid symbols represent the presence, of 100 nM pyridoxal. In panel (B) the *pdxJ* mutant carried a plasmid expressing *SNZ3*. In addition, the strain had a compatible empty vector (triangles), or a plasmid expressing p*SNO3* (diamonds). All strains were grown on NCN minimal medium with glycerol and glutamine (*i.e.*, low ammonia). Open symbols represent growth in the absence, while solid symbols represent the presence, of 100 nM pyridoxal. Growth was monitored as a function of optical density at 650 nm with shaking at 37 °C. Error bars indicate the standard deviation of three independent biological replicates.

The *SNZ/SNO* pathway for PLP synthesis is less complex than the multi enzyme DXP-dependent pathway used by *S. enterica* and other organisms. If functional, introduction of these enzymes into *S. enterica* would i) allow characterization of single paralogs, and ii) provide a heterologous system that could be used to probe *S. enterica* with a simplified metabolic network. A plasmid expressing *SNZ3* from the *lac* promoter on pSU18 (pDM1595) was introduced into a *S. enterica* strain lacking the pyridoxine-5′-phosphate (PNP) synthase (E.C. 2.6.99.20) encoded by *pdxJ* (Figure 1). *SNZ3* provided *in trans* supported growth of the resulting strain in minimal (NCE) media with glycerol as a carbon source. These data showed that *SNZ3* was necessary and sufficient to synthesize PLP in the *pdxJ* mutant of *S. enterica* (Figure 6A), and were generally consistent with the ability of *SNZ1* from *Cercospora nicotianae* to complemented a *pdxJ* strain of *E. coli* (Wetzel *et al.* 2004).

NCE medium has high levels of ammonia that could bypass a need for the putative glutaminase activity of Sno3p. In fact, *SNZ3* failed to allow growth of a *pdxJ* mutant when glutamine (1 mM) was provided as sole nitrogen source (Figure 6B). When pDM1595 was present and *SNO3* was provided on a compatible plasmid (pDM1596), growth of the *pdxJ* mutant was restored to the level allowed by exogenous pyridoxal. In total, these data showed that *SNZ3* encodes a functional PLP synthase and that *SNO3* is needed only when ammonium concentrations are low.

### Snz3p has PLP synthase activity in vitro

**Figure 7:**
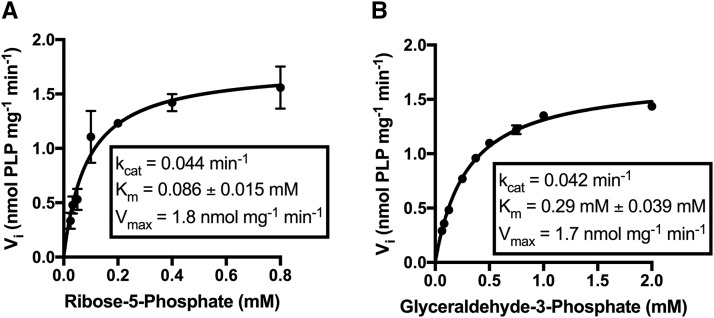
Snz3p is a PLP synthase. Saturation curves for Snz3p were determined by measuring the initial rate of PLP formation *vs.* ribose-5-phosphate (A) or D/L-glyceraldehyde-3-phosphate (B) as a substrate. Reactions were performed in 50 mM Tris pH 8.0 at 37 °C, containing 85 µM Snz3p. When ribose-5-P was titrated, the reaction mix contained 2 mM D/L-glyceraldehyde-3-phosphate and 20 mM NH_4_. When D/L-glyceraldehyde-3-phosphate was titrated, the reaction mix contained 1 mM ribose-5-phosphate and 20 mM NH_4_. All reactions were performed in triplicate, and error bars are shown.

The PLP synthase activity of *SNZ3* suggested by sequence identity and the *in vivo* data above was confirmed *in vitro*. The *SNZ3* gene was cloned into a pTEV5 vector and His_6_-Snz3p was purified to >95% homogeneity prior to cleaving the His_6_ Tag to generate native protein. The purified protein was assayed for PLP synthase activity *in vitro*. An assay reaction with ammonium sulfate, glyceraldehyde-3-phosphate and ribose-5-phosphate as substrates was used to define basic kinetic parameters of Snz3p. The K_m_ for ribose-5-phosphate was 0.09 ± 0.02 mM while the K_m_ for glyceraldehyde-3-phosphate was 0.29 ± 0.03 mM (Figure 7). The K_cat_ for ribose-5-phosphate was 0.044 min^-1^ while the K_cat_ for glyceraldehyde-3-phosphate was 0.042 min^-1^. These data were similar to those reported for Snz1p (K_m_ = 0.11 mM and 0.3 mM, K_cat_ = 0.036 min^-1^ and 0.039 min^-1^ for ribose-5-phosphate and glyceraldehyde-3-phosphate, respectively), when assayed in the absence of Sno1p (Neuwirth *et al.* 2009) . Kinetic constants available for the *Bacillus subtilis* PLP synthase in the absence of the glutaminase subunit demonstrate that while this synthase has a higher affinity for ribose-5-phosphate and glyceraldehyde-3-phosphate (K_m_ = 0.068 mM and 0.077 mM, respectively), its catalytic turnover is similar (K_cat_ = 0.02 min^-1^) (Raschle *et al.* 2005).

### Conclusions

*SNZ3* encodes a functional PLP synthase that uses glyceraldehyde-3-phosphate, ribose-5-phosphate and ammonia as substrates. Despite the near identical kinetic constants of Snz1p and Snz3p, results here demonstrate the two isozymes have different roles *in vivo*. The data showed that *SNZ2* or *SNZ3*, but not *SNZ1*, was sufficient to generate PLP for growth on MVD medium (*i.e.*, in the absence exogenous PN or Thi). *SNZ1* supported growth only when PN and/or thiamine were provided. This result suggested that *SNZ1* was unable to satisfy the PLP requirement for thiamine synthesis. The finding that *SNZ2* and/or *SNZ3* are important for thiamine, specifically HMP, synthesis supports a connection between *SNZ2/3* and the Thi5p family of enzymes. The poorly characterized Thi5p enzymes use PLP as a substrate rather than a co-factor to generate the HMP-P moiety used for thiamine synthesis (Lai *et al.* 2012; Coquille *et al.* 2012). The finding that the lack of a specific *SNZ* paralog impacts thiamine biosynthesis, out of many metabolic pathways that use PLP as a cofactor, suggests that there are unique requirements for PLP in this pathway, likely involving the Thi5p family of enzymes.

To our knowledge these data provided the first evidence of distinct roles for the *SNZ* paralogs *in vivo* that was not due to regulation of gene expression. It is worth noting that the phenotypes key to the above conclusions were not obvious from past studies using dropout media (Rodríguez-Navarro *et al.* 2002; Stolz and Vielreicher 2003). In fact, the previous studies led to the conclusion that *SNZ1* encoded the primary PLP synthase, a conclusion that the results herein bring into doubt. Although not conclusive from the results with *S. cerevisiae*, studies with the heterologous host *S. enterica* showed that the glutaminase subunit *SNO3* is dispensable for PLP synthesis in the presence of excess ammonia. These data further showed that the DXP-dependent pathway for PLP synthesis could be replaced by a single gene (*SNZ3*) in *S. enterica*, and defined a heterologous system that will be valuable in studies to probe network structure with a simplified B_6_ metabolism.
